# Collaboration on antimicrobial stewardship practices amongst university health systems, Veterans Affairs medical centers, and other affiliates: opportunities for greater harmony

**DOI:** 10.1017/ash.2023.495

**Published:** 2023-12-01

**Authors:** Matthew M. Hitchcock, J. Daniel Markley, Daniel Tassone, Meghan Kamath, Kimberly B. Lee, Adam Greenfield, Barry Rittmann, Sangeeta Sastry

**Affiliations:** 1 Department of Medicine, Division of Infectious Diseases, Central Virginia VA Health Care System, Richmond, VA, USA; 2 Department of Internal Medicine, Division of Infectious Diseases, Virginia Commonwealth University School of Medicine, Richmond, VA, USA; 3 Central Virginia VA Health Care System, Richmond, VA, USA; 4 Department of Clinical Pharmacy, Virginia Commonwealth University Health System, Richmond, VA, USA

## Introduction

Trainees and faculty often rotate through multiple clinical sites, which may include university medical centers, community hospitals, and Veterans Affairs medical centers (VAMCs), each with their own antimicrobial stewardship program (ASP). Education on appropriate antimicrobial prescribing can be highly variable with trainees and staff having disparate guidance and varied interactions with ASPs at different locations.^1^ Affiliated academic health systems should have a particular interest in ASP collaboration across sites as they serve as the training ground for healthcare professionals. Collaboration can improve the consistency of recommendations and facilitate growth of ASPs through the sharing of resources. However, collaboration can be challenging due to site-specific differences. Each ASP must tailor its initiatives to local needs, which complicates collaborative efforts when standardization of practices is overemphasized.

Herein, we propose applying the concept of harmony to collaborative stewardship efforts. Harmony is not uniformity but emerges when unique entities work collectively towards a common goal while remaining distinct, like the notes of a chord.^2^ Harmonious collaboration involves multiple stakeholders working together to share information and practices, identify performance gaps, and develop collective solutions.^3^ This requires synergistic alliances that seek contributions from all parties and respect different perspectives.^4^ Stewardship initiatives within the Veterans Health Administration (VHA) can serve as a model for harmonious collaboration within affiliate academic networks.

## The VHA ASP model

Formal affiliations between VAMCs and academic hospitals were established in 1946, with the goals of supporting the medical care of the veteran population, expanding the national healthcare workforce, and improving medical practice through education and research.^5^ Currently, VHA is the second largest funding source for graduate medical education, its facilities provide clinical sites for thousands of trainees from university programs, and many faculty have dual appointments with VHA and affiliate academic medical centers.^6,7^

In 2010, the VHA began the Antimicrobial Stewardship Initiative to provide national guidance and resources for the development of VAMC ASPs.^8,9^ The initiative is implemented through the Antimicrobial Stewardship Task Force (ASTF), a multidisciplinary team that develops educational tools, sample policies, antimicrobial utilization dashboards, and other national resources.^8,9^ The ASTF facilitated the creation of regional stewardship collaboratives organized within the 21 Veterans Integrated Service Networks.^9^ While it provides tools and resources nationally, the VHA has not mandated specific stewardship initiatives locally. Local ASP policies and initiatives informed by nationally developed tools and resources have effectively reduced inpatient and total antibiotic use by 12% and 2.1%, respectively, in VHA studies.^8,10^

## Opportunities for harmonization

There are several ways ASPs across affiliated sites could harmonize their programs (Table 1). Development of treatment guidance by ASPs remains a priority intervention within the Centers for Disease Control and Prevention Core Elements of Antibiotic Stewardship, and collaborative guideline development ensures that nuances such as formulary differences and local resistance patterns are addressed.^11–13^ A recent example is the Colorado Hospital Association’s antimicrobial stewardship collaborative, which implemented evidence-based guidelines for urinary tract infections (UTIs) and skin and soft tissue infections (SSTIs) at 26 hospitals, though this did not include a VAMC (personal correspondence).^12^ Over an 18-month period, there were significant reductions in the use of fluoroquinolones for UTIs, broad-spectrum antibiotics for SSTIs, and total duration of antimicrobial therapy at the participating hospitals.^12^ Treatment guidelines can be made available via mobile-based medical applications to facilitate access and increase adherence.^13–15^

**Table 1.** Opportunities for harmony between ASPs within University Health Systems, Veterans Affairs Medical Centers, and Other Affiliates

Topics for Collaboration	Practical Implementation
System-wide infectious disease treatment guidance	Co-author local antimicrobial treatment guidance with input from ASPs across the system. Disseminate guidance via shared mobile-based medical application accessible to all trainees, faculty, and staff.
Regional antibiogram	Construct a regional antibiogram from facility-level data that can be shared across systems.
Educational materials and activities	Collaboratively develop educational initiatives and collectively implement across affiliated sites. Examples include: • Urine culture diagnostic stewardship • Management of acute respiratory infection Develop a cohesive ASP curriculum for trainees. Provide stewardship lectures to trainees and staff across systems.
Programmatic development through mentorship and sponsorship	Provide regular opportunities for mentorship and sponsorship within the affiliated network to encourage faculty and programmatic development. Examples include: • ASP network planning sessions • Multi-center ASP lecture series, case conferences, and/or journal clubs • Provide opportunities to give lectures and educational sessions throughout the network
Research activities	Develop collaborative system-wide research projects. Examples include: • Impact of collaboratively developed guidelines • Diagnostic stewardship interventions • Implementation of cascade and selective antimicrobial susceptibility reporting

Note. ASP, antimicrobial stewardship program.

Another opportunity for harmonization is the development of regional antibiograms to provide useful epidemiologic information on antimicrobial resistance, especially for smaller facilities that may have insufficient isolates to produce a validated local antibiogram.^16,17^ The information can be specifically helpful for academic networks that frequently share patients.

Perhaps the greatest opportunity for harmonization is the production of educational resources for the affiliated academic network. These include teaching sessions for trainees and staff, which take considerable time to produce and are often duplicated across sites. Sharing these resources can reduce the burden for a smaller ASP, offer additional opportunities for professional development, and allow for shared trainees to receive a more cohesive curriculum across clinical sites. Antimicrobial stewardship programs can also develop joint educational conferences, including case discussions, lectures, and/or journal clubs. These can provide further opportunities to discuss current literature and ways to implement evidence-based practices locally. Collaborative efforts can be formalized in periodic strategic planning sessions between affiliated ASPs to develop common goals and initiatives. Mentorship and sponsorship from more experienced ASPs can facilitate the growth and development of staff at resource-limited or newer programs. An emphasis on a shared mission is critical to foster growth and promote sharing of existing resources.

Overall, there is a paucity of literature regarding stewardship integration across academic or looser affiliated networks. Most of the published literature focuses on partnerships between ASPs and specific institutional service lines or large, more formalized networks such as centralized, health-system ASPs, consultative stewardship services, and hospitals with shared EMRs.^18–23^ This provides a significant opportunity to further the literature on collaborative stewardship interventions in affiliated networks.

## Present challenges

While there are opportunities for ASP harmonization within affiliated systems, many challenges remain. Antimicrobial stewardship programs may have insufficient resources, expertise, and support to carry out their activities effectively.^24^ There may be drastic differences in programmatic structure, antimicrobial restriction policies, pharmacy formularies, as well as the overall culture between facilities. The benefits of harmonization are typically downstream, while the start-up cost, and time commitments, can be high, especially for programs already stretched to complete their core tasks. Collaborative guideline development introduces additional complexities to production, approval, and revision as compared to single-facility guidance. Collaboratively developed guidelines take longer to produce and require facility-level approval for the initial document and any revisions. This creates obstacles to keeping the guidance documents updated but can be managed by ensuring appropriate stakeholders are involved in initial development and periodic review. Antimicrobial stewardship programs can also make supplemental guidance while remaining a party to the collaborative products.

Partnerships between VAMCs and academic affiliates can be uniquely challenging due to additional federal privacy protections on patient-level data and the VA EMR, which create logistical hurdles for research, especially clinical decision support interventions. Veterans Affairs medical centers also have a unique patient population that is generally older and predominantly male, which differs from those at the affiliates.^25^ Collectively, these barriers, along with the existing VA stewardship infrastructure, make it easier for VAMCs to collaborate within the VA network rather than with their academic affiliates, creating parallel rather than integrated networks.

## Conclusion

Antimicrobial stewardship programs within an affiliated academic network have common goals and should seek practical ways to harmonize their efforts. The VHA has served as a leader in the implementation of antimicrobial stewardship in a complex network and health systems affiliated with VAMCs should take advantage of this expertise. The goal of collaboration within these networks should be harmonization of education and practices, not standardization. While standardization within an organization improves the quality and safety of care,^26^ an overemphasis on standardization between different facilities serves as a barrier to collective work. Local ASPs operate in specific contexts, and differences in EMRs, formularies, patient populations, clinical laboratory capabilities, and overall resources must be acknowledged. Harmonious collaboration should emphasize common goals and interventions that can be reasonably achieved, particularly around improving the consistency of education and stewardship recommendations for shared trainees and staff. The overuse of antibiotics requires collective action to address,^27^ and this begins in the networks that bind us.
